# Metabolite quantification of faecal extracts from colorectal cancer patients and healthy controls

**DOI:** 10.18632/oncotarget.26022

**Published:** 2018-09-07

**Authors:** Gwénaëlle Le Gall, Kiran Guttula, Lee Kellingray, Adrian J. Tett, Rogier ten Hoopen, Kate E. Kemsley, George M. Savva, Ashraf Ibrahim, Arjan Narbad

**Affiliations:** ^1^ Quadram Institute Bioscience, Norwich Research Park, Norwich, UK; ^2^ Division of Molecular Histopathology, Department of Pathology, University of Cambridge, Cambridge, UK; ^3^ Centre for Integrative Biology, University of Trento, Trento, Italy

**Keywords:** NMR, colorectal cancer, markers, metabolite, metabolomics

## Abstract

Colorectal cancer (CRC), a primary cause of morbidity and mortality worldwide is expected to rise in the coming years. A better understanding of the metabolic changes taking place during the disease progression is needed for effective improvements of screening strategies and treatments. In the present study, Nuclear Magnetic Resonance (NMR) metabolomics was used to quantify the absolute concentrations of metabolites in faecal extracts from two cohorts of CRC patients and healthy controls. The quantification of over 80 compounds revealed that patients with CRC had increased faecal concentrations of branched chain fatty acids (BCFA), isovalerate and isobutyrate plus valerate and phenylacetate but diminished concentrations of amino acids, sugars, methanol and bile acids (deoxycholate, lithodeoxycholate and cholate). These results suggest that alterations in microbial activity and composition could have triggered an increase in utilisation of host intestinal slough cells and mucins and led to an increase in BCFA, valerate and phenylacetate. Concurrently, a general reduction in the microbial metabolic function may have led to reduced levels of other components (amino acids, sugars and bile acids) normally produced under healthy conditions. This study provides a thorough listing of the most abundant compounds found in human faecal waters and presents a template for absolute quantification of metabolites. The production of BCFA and phenylacetate in colonic carcinogenesis warrants further investigations.

## INTRODUCTION

The global burden of colorectal cancer (CRC), which accounted for about 1.4 million new cases and almost 700 000 deaths in 2012, is expected to rise by 60% by 2030 [1]. Although the incidence is decreasing in countries with high human development index (HDI) where around two-thirds of cases and deaths worldwide are occurring, rapid increases in incidence and mortality are now seen in many medium HDI countries [1, 2]. The risk of colorectal cancer increases with age, high consumption of red or processed meat or alcohol, low intake of fruit and vegetables, smoking, high body mass index and low physical activity [2]. Early detection is key to a favourable 5-year survival rate [3]. Screening programs in many countries are based on a combination of non-invasive clinical markers (faecal occult blood and faecal immunochemical tests) and endoscopic techniques (flexible sigmoidoscopy, colonoscopy and computed tomographic colonography) but due to the variable sensitivity of existing non-invasive tests, new non-invasive procedures are still urgently needed. DNA-based stool [4] and serum-based tests such as carcinoembryonic antigen and carbohydrate antigen 19-9 [5, 6] hold promise but low sensitivity remains an issue. The widespread use of colonoscopy has resulted in a significant decrease in the mortality of colorectal cancer due its high rate sensitivity [7] but is invasive, costly and associated with bleeding and perforation.

Metabolomic analysis consists of measuring and comparing the levels of metabolites across samples to discover potential biomarkers. This non-invasive approach has been applied to tumour and adjacent tissue [8–9], blood plasma [10–12], urine [13, 9] and faecal extracts [14–20] to search for markers of early diagnosis and for staging of CRC. The commonality in the biopsy studies was an increase in amino acids and lactate in tumour tissue [8–9]. Changes in plasma partly mirrored those findings since amino acid levels differed [10–12] and lactate levels were found to be higher in CRC samples [10]. Additionally, intermediates of purines, pyrimidines and the tricarboxylic acid (TCA) cycle were altered [11–12]. Variation in amino acids and TCA cycle pathways was also observed in urine [13]. The studies on faecal metabolomics reported changes in short chain fatty acid (SCFA), amino acid, and lipid metabolism. Butyrate levels were depleted in many of the studies [14, 16, 19–20] but with one exception [18]. Similarly, conflicting results emerged on acetate with three studies reporting an increase in concentration [16, 18, 20] while two others stated a decrease [14, 19]. A more consensual trend of elevated amino acid levels was found for most studies [14, 16, 20] except for two studies that reported a diminution of the level of glutamine [18–19]. Results from metabolomics studies typically do not use absolute quantitation since the primary aim with this approach is to provide a rapid screening for group comparisons. The novelty in the present study is two-fold: firstly, the provision of a full list of quantified faecal metabolites in a healthy human and secondly, detecting the metabolic differences between healthy individuals and two independent cohorts of CRC patients. Absolute quantification of faecal SCFA and amino acids has previously been published [21–22] but reports on the amounts of organic acids, sugars, nucleosides, and other molecules present in human faeces are scarce. Establishing the composition and the expected quantity of compounds in faecal extracts would help to clarify the role of faecal metabolites in the development of CRC and other gastrointestinal tract diseases. In this study, we analysed the faecal microbiome of one cohort and the metabolomes of two cohorts of CRC patients and healthy controls and have identified quantifiable differences in the composition and function of the gut microbiomes of CRC patients.

## RESULTS

### Patient demographics and study design

The first set of samples from 20 CRC patients and 20 healthy controls was analysed in 2012. The second cohort consisting of 30 CRC patients and 30 healthy individuals were analysed two years later. Hence 50 age and sex-matched pairs of stool samples were used for ^1^H NMR profiling of faecal metabolites. One outlier was excluded from the second set due to poor spectral quality. Metagenomics analysis was also applied to the faecal microbiome of the first set (*n* = 40). Patients and tumour characteristics are outlined in Table 1.

**Table 1:** Patient demographics and tumour characteristics

		set 1		set 2	
Patients		Colorectal cancer	Healthy	Colorectal cancer	Healthy
		*N* = 20	*N* = 20	*N* = 30	*N* = 29
Age, years	Mean	67	67	66	66
	Range	61–72	60–74	60–74	60–74
Sex	Women	8	8	9	9
	Men	12	12	21	21
Tumour site	caecum	2		3	
	ascending	2		2	
	transversal	3		0	
	descending	1		2	
	sigmoid	4		17	
	rectum	6		6	
Cancer size, mm	Mean	35		24	
	Range	12–70		15–40	
Dukes's stage	A	3		3	
	B	7		2	
	C	5		11	

### Faecal metabolite quantification

**Figure 1:**
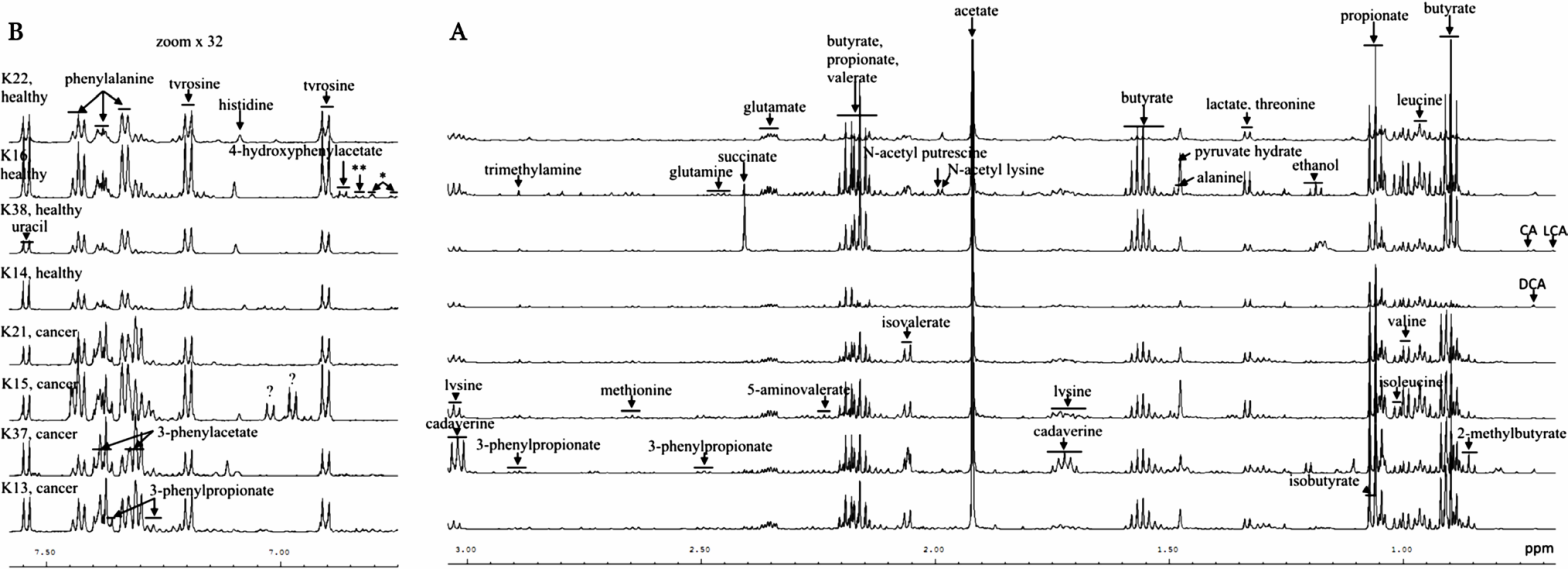
Typical 600 MHz ^1^H NMR spectra of aqueous faecal extracts from 4 CRC patients and age and sex matched controls High and mid (**A**) and low (**B**) field regions of the 1H NMR spectra. Key: ^*^, 3-hydroxyphenylpropionate; ^**^ p-cresol.

The faecal ^1^H NMR spectra were dominated by signals arising from the three main SCFA namely, acetate, propionate, and butyrate and characterised by low levels of many other metabolites (Figure 1A). Over 80 compounds were identified with 2-dimensional NMR experiments, the literature data [18, 23] and the human metabolome database and quantified in an absolute manner (Supplementary Table 1). Compounds included energy related metabolites such as fatty, organic, and amino acids, sugars, osmolytes, amines, alcohols, phenolic compounds, nucleobases, nucleosides, nucleotides, vitamin B3 and bacterial degradation products. Findings prior (Supplementary Table 2) and after (Supplementary Table 1) combining the two sets of data did not differ substantially.

### Biomarkers of CRC

Visual inspection indicated that a subset of cancer profiles was characterised by high levels of isovalerate, isobutyrate and phenylacetate (Figure 1A and 1B). Principal component analysis (PCA) of the samples from both sets showed overlapping but some separation between cancer and healthy groups (Supplementary Figure 1). However, a large number of principal components were needed to account for a significant proportion of variation in the dataset.

**Figure 2:**
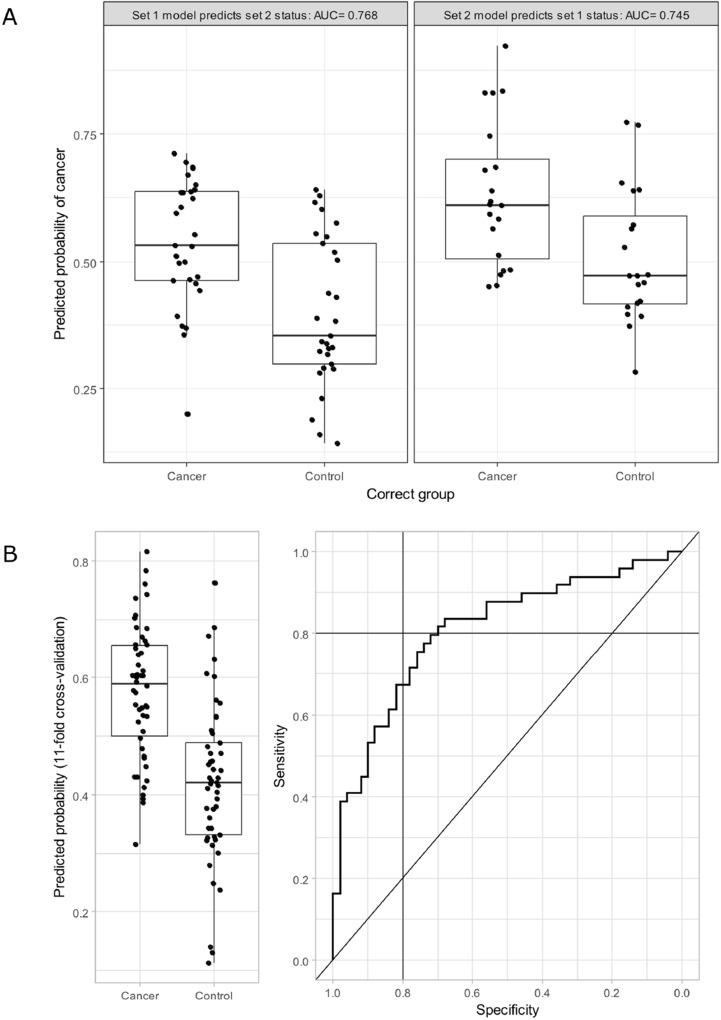
(**A**) The predicted probability of cancer estimated by PLS-DA predictive power using each set and validated by applying to the other. AUC = area under the receiver operator characteristic (ROC) curve, and reflects the probability that a randomly selected CRC patient has a higher predicted probability of cancer than a randomly selected control. (**B**) Left panel shows the predicted probability of cancer estimated by scaled PLS-DA models using Box-Cox transformed metabolite concentrations, stratified by cancer status. 11-fold cross-validation was used, hence each predicted probability is estimated independently of the true cancer status of the patient. Right hand panel shows the ROC curve estimated using the same data (AUC = 0.8) with solid lines indicating sensitivity and specificity of 80%.

Conversely, with PLS-DA, cross-validation within each set suggested that using two components was optimal for prediction. When two components models were estimated in each set and applied the predictive power was similar in each case (Figure 2A). Using 11-fold cross-validation within the combined set led to an C-statistic of 0.80 (Figure 2B). A sensitivity of 80% was achieved at a specificity of 70%, while specificity of 80% could be achieved with sensitivity of 67%. With a threshold of 50% used to identify cases from controls, the PLS-DA model classified 74 (75%) of 99 cases correctly. This suggests that while it is not possible to completely identify CRC from control patients using faecal metabolites a reasonable degree of discrimination is possible, even within a cohort that has screened positive using FOBt.

In univariate analysis, twenty metabolites had FDR-adjusted *p*-value lower than 0.05 when values were compared between cancer patients and controls. Fifteen had significantly lower levels among cancer patients, five had significantly higher levels (Table 2). Distributions of each metabolite concentration in which a significant difference was observed (at FDR < 0.05) are shown in Supplementary Figures 2 (raw concentrations) and 3 (Box-Cox transformed).

**Table 2:** The ratio of metabolite concentrations between CRC patients and controls

Metabolite	Mean concentration ratio (CRC/control)	*t*-statistic	*p*-value (*t*-test)	*p*-value (FDR adjusted)
Lower among cancer patients				
Cholate	0.13	−5.06	0.00000	0.0002
Taurine	0.39	−4.59	0.00001	0.001
Glutamine	0.67	−4.24	0.00005	0.002
ß-Alanine	0.35	−4.13	0.00008	0.002
Glucose	0.38	−3.91	0.00018	0.003
Lithodeoxycholate	0.01	−4.02	0.00015	0.003
Xylose	0.38	−3.31	0.00134	0.012
Deoxycholate	0.44	−3.28	0.00145	0.012
Ornithine	0.59	−3.33	0.00124	0.012
Glycerol	0.68	−3.20	0.00189	0.015
Guanosine	0.21	−3.16	0.00248	0.018
Isoleucine	0.70	−3.01	0.00339	0.021
Methanol	0.62	−2.77	0.00679	0.040
Galactose	0.61	−2.66	0.00913	0.048
4-Aminohippurate	0.39	−2.61	0.01058	0.050
Higher among cancer patients				
Isovalerate	1.75	3.59	0.00052	0.007
Hexose-phosphate^*^	2.02	3.37	0.00107	0.012
Phenylacetate	1.73	3.01	0.00335	0.021
Isobutyrate	1.54	2.74	0.00741	0.041
Valerate	1.42	2.62	0.01029	0.050

T-statistics and *p*-values are calculated using Box-Cox transformed concentrations. Adjusted *p*-values are calculated using the false discovery rate method of Benjamini and Hochberg to correct for the large number of hypotheses being tested.

^*^signal at 5.61 ppm, most likely glucose- or galactose-1-phosphate.

While not all identified metabolites show statistically significant differences in both sets of patients, ratios of effects are largely consistent across sets. The distribution of *p*-values comparing the differences in effects across sets was uniform, suggesting that there was no difference between sets with respect to associations between metabolites and cancer status (Supplementary Table 2). Some of the markers were unique to a set (succinate for set 1, p-cresol for set 2) but the majority showed a consistent trend of increased or decreased levels in both sets, although for each circumstance the metabolite had a non-significant *p* value in one of the sets.

Table 2 shows the ratios of concentrations of metabolites that are statistically significantly higher or lower between groups (at FDR corrected *p*-value < 0.05). Cancer patients are characterised by higher concentrations of iso short-chain fatty acids (valerate, isobutyrate, isovalerate), phenylacetate and, a sugar-phosphate whose signals arise at 5.61 ppm, and lower concentrations of methanol, amino acids (glutamine, ornithine, isoleucine, taurine, and b-alanine), sugars (glucose, galactose and xylose), and bile acids (deoxycholate, lithodeoxycholate and cholate). There is some evidence for differences in many other metabolites with individual *p* values of less than 0.05 although their statistical significance may arise through the large number of hypotheses being tested. (Supplementary Table 1). Correlations between selected metabolites are shown in Supplementary Figure 4. The highest levels of isoacids, phenylacetate and phenylpropionate were all associated with the same five cancer patients.

### Gut microbiota composition of set 1

**Figure 3:**
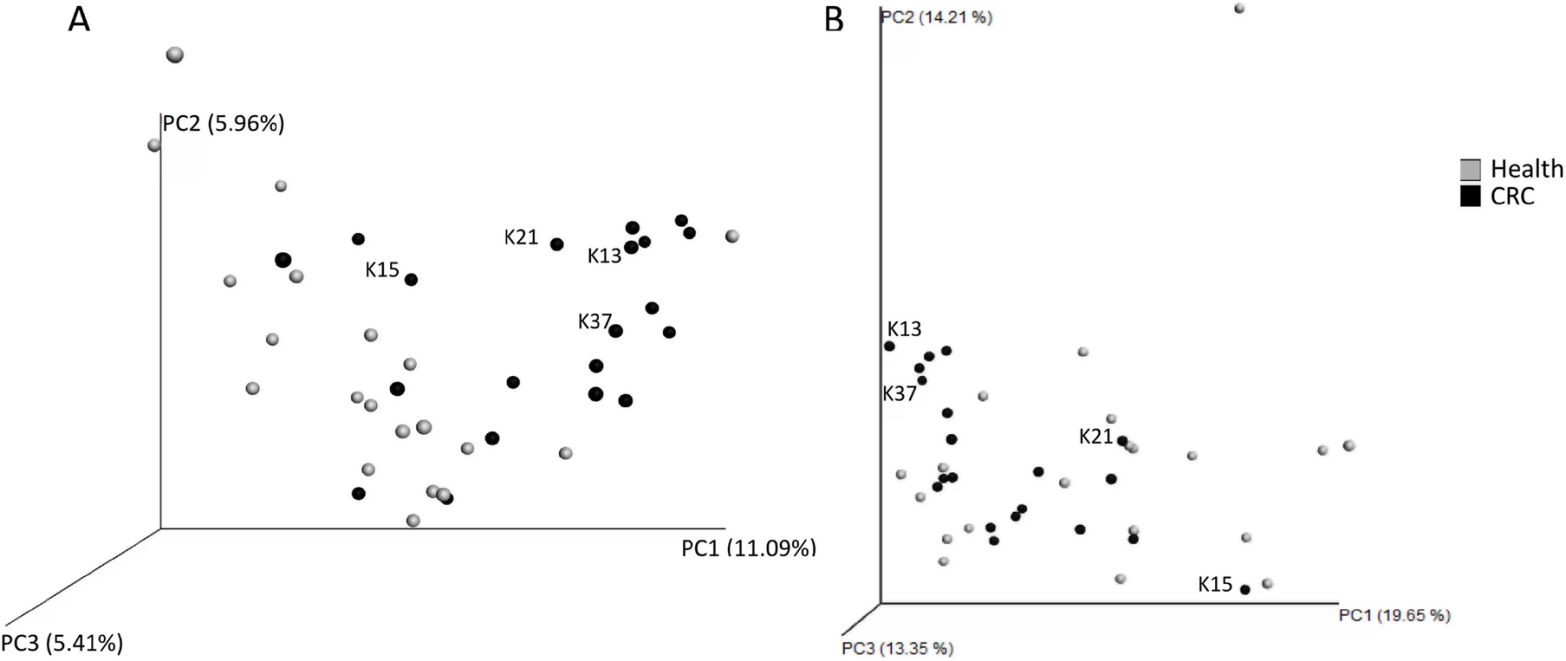
Beta-diversity analysis of faecal microbiota of healthy controls (grey) and colorectal cancer patients (black) The data-points associated with the subset of CRC patients (K13, K15, K21, & K37) identified from the metabolomic analyses are labelled. (**A**) unweighted beta-diversity analysis and (**B**) weighted beta-diversity analysis were performed using the Unifrac metric in QIIME 1.9.1, and visualised as 3D principal coordinates analysis plots using Emperor.

The gut microbiota composition of the first cohort (20 CRC patients versus 20 age and sex matched healthy controls) was assessed using 454 pyrosequencing and the QIIME pipeline. This analysis produced 167 572 sequence reads, with an average of 4189 ± 1476 reads per sample, which clustered into 5762 operational taxonomic units at 97% identity. PCoA plots were generated to investigate whether the microbiota of patients with CRC were more similar to one another than those obtained from healthy controls. The unweighted analysis, whose results are based on which taxa are shared between samples, suggested a difference in the composition of the microbiota between the two groups (Figure 3A). However, these differences were less clear following the weighted analysis, which also takes the relative abundances of taxa into consideration (Figure 3B). At the family level it was observed that, on average, CRC patients had a larger relative abundance of Ruminococcaceae (32.65% ± 8.72% vs 20.35% ± 13.34% (*P* = 0.001)) and a lower proportion of Lachnospiraceae (30.34% ± 11.49% vs 42.57% ± 18.33% (*P* = 0.016)). Of the seven bacterial families that were significantly different between CRC patients and healthy controls, five are members of the Clostridiales order (unclassified Clostridiales (*P* = 0.027), Christensenellaceae (*P* = 0.002), Mogibacteriaceae (*P* = 0.017), and Lachnospiraceae & Ruminococcaceae), as well as Porphyromonadaceae (Bacteroidales) and an unclassified family member of the order RF39 (*P* = 0.006) (Table 3). Interestingly, although only present as a small proportion of the microbiota, the Archaeal family Methanobacteriaceae were found in 50% (10/20) of CRC patients compared to 10% (2/20) of healthy controls (*P* = 0.009). Bar charts depicting the taxonomic composition of each faecal sample at the genera level did not suggest a community profile that may be a signature of CRC (data not shown). However, some statistically significant differences in the relative proportions of certain genera were observed between the two groups, with 14 of the 17 taxa identified found at a higher proportion in the CRC samples (Table 3). Further statistical analysis of the microbiota composition of the subset of CRC patients, which were identified as outliers through metabolomic analyses, produced a list of taxa that were present at significantly different proportions to the remaining CRC cohort (Supplementary Table 3).

**Table 3:** Statistically significant taxa that differ between healthy and colorectal cancer patients

Microbial taxa	Healthy (%)	CRC (%)	*P* value
o_Clostridiales	2.14 ± 2.96	4.66 ± 4.14	0.027
o_RF39	0.20 ± 0.55	1.95 ± 2.26	0.006
o_Clostridiales; f_Ruminococcaceae	1.29 ± 1.62	3.76 ± 2.47	0.001
f_Christensenellaceae	0.12 ± 0.31	0.81 ± 0.75	0.002
f_Mogibacteriaceae	0.13 ± 0.14	0.37 ± 0.26	0.002
f_Coriobacteriaceae	0.20 ± 0.19	0.58 ± 0.49	0.001
f_Erysipelotrichaceae;g_Clostridium	0.45 ± 1.07	0.05 ± 0.15	0.044
f_Ruminococcaceae;g_Ruminococcus	5.98 ± 6.70	11.40 ± 5.23	0.015
g_Methanobrevibacter	<0.01 ± 0.03	0.10 ± 0.15	0.01
g_Parabacteroides	1.47 ± 1.77	0.62 ± 0.52	0.014
g_Clostridium	0.37 ± 0.53	0.05 ± 0.07	0.012
g_Peptostreptococcus	<0.01 ± 0.01	0.05 ± 0.08	0.029
g_Anaerofilum	<0.01 ± 0	0.03 ± 0.05	0.011
g_Oscillospira	1.23 ± 1.07	2.64 ± 1.89	0.006
g_Sporobacter	0 ± 0	<0.01 ± 0.01	0.045
g_Eubacterium	1.58 ± 3.01	4.21 ± 6.21	0.049
g_cc_115	<0.01 ± 0.01	0.07 ± 0.16	0.03

Preceding letter indicates taxonomic level: o = order; f = family; g = genus. Values shown are mean ± SD.

A canonical correlation analysis on the metabolite data and the matching microbiota data I set 1 (Supplementary Figure 5) showed there was a weak but significant link between the two microbiota and NMR data sets but no separation between the two groups of samples (cancer vs controls). No good correlation was found in the heap map correlating individual metabolite with individual microbial trait (not shown).

## DISCUSSION

In the present study, we have presented a comprehensive list of faecal metabolites expressed in concentration units among 50 CRC patients and 49 controls, recruited through a national screening programme who had screened positive using FOBt.

NMR quantification, an approach widely applied to urine and blood samples [24, 25] has not yet been applied to faecal water extracts. We have used our expertise in metabolite identification of faecal waters by ^1^H NMR [26] to prepare a thorough list and used a specialised software to quantify the metabolites detectable in faecal extracts.

Previous faecal metabolomics studies on CRC have qualitatively detected metabolites such as short chain fatty acids and amino acids [14–20], but to our knowledge this is the first time that faecal absolute concentrations in healthy and cancer patients have been determined. The concentrations of SCFA, amino acids, lactate, phenol, p-cresol, and bile acids are consistent with those reported in the literature [21–22, 27, 28]. The absolute concentration of the other compounds has not been reported before. Dietary polysaccharides/fibre that reach the large intestine are broken down by bacteria into end-products such as SCFA, mainly acetate, propionate, and butyrate, lactate, ethanol, methane, hydrogen, and CO_2_ [29]. Colonic health is associated with a diet rich in non-digestible starch and is reflected by fairly high levels of butyrate, acetate, and propionate [30–31]; however, the presence/absence of other elements from the metabolite composition also contribute to the maintenance of a healthy gut [29–31]. Host derived glycans notably mucins, dietary amino acids and proteins are other major nutrient sources for gut bacteria [29, 32]. In this study, findings from two independent sets of CRC patients and healthy controls consistently showed an elevation of isovalerate, isobutyrate, valerate and phenylacetate levels in CRC and a diminution in the concentrations of amino acids, sugars and methanol.

Two previous studies have reported an increase of isovalerate, isobutyrate and valerate levels in CRC faecal extracts [16, 20], one study reported a decrease [33] and another one no differences [34]. Straight-chain SCFA (butyrate, propionate, acetate and valerate) are products of saccharides and amino acids while branched SCFA (BCFA), isobutyrate, isovalerate and 2-methylbutyrate are specifically attributed to the degradation of branched amino acids (BAA, valine, leucine and isoleucine) [35]. In the past, BCFA and valerate have been associated with an augmented risk of developing CRC principally because protein fermentation has been tied in with a high protein intake [29]. However, contrary to other microbial and chemical products such as hydrogen sulphide, p-cresol, phenol, haem iron, N-nitroso compounds, polycyclic aromatic hydrocarbons, and heterocyclic amines which are reported to be detrimental [29, 31, 36], the isoacids although indicative of putrescible fermentation, are not associated with cell toxicity [35]. The World Health Organization recently classified red meat and processed meat as carcinogens however no mechanisms or causal link have yet been established [36]. Additionally, a recent publication showed an increase in faecal BCFA after a high protein intake but no toxicity [37]. Moreover, the presence of a small amount of branched SCFA and valerate (2–4 mmol/kg) is of normal occurrence in healthy adult individuals [38] and neonates [39]. Increased transit time in the GI tracts has also been associated with a high concentration of putrefactive products [27]; however contrary to BCFA, the levels of putrescine, N-acetylputrescine, cadaverine and p-cresol were not increased suggesting no enhanced protein putrefaction in our study. Interestingly, Andrieux and colleagues [40] proposed an alternative source of BCFA production. They reported an increase in isobutyrate and isovalerate levels with age and attributed the differences to a change in bacterial mucin degradation. Concomitantly, a high degree of correlation exists between the levels of isobutyrate and isovalerate regardless of the host species [21, 41] which suggests the presence of a universal substrate. In line with those findings our data showed strong correlations between isobutyrate and isovalerate, isobutyrate and valerate and isovalerate and valerate (Supplementary Figure 4). There are two main sources of endogenous microbial substrate: intestinal sloughed cells and host glycan mucins [40, 41]. Production of BCFA from sloughed cells is a plausible event since intestinal tissue is particularly rich in BAA [42]. It is also tempting to speculate that changes in microbial mucolytic activity could occur in CRC since an increase in abundance of mucinophilic bacteria (*Fusobacterium nucleatum and Akkermansia spp*) has recently been reported [31, 43]. A causal link has not yet been determined [43] but opportunistic mucin degradation is a theory that has been proposed [44]. Proline and BAA are abundant constituents of human mucin [45], thus microbial mucolytic activity followed by the fermentation of proline, valine and leucine could contribute to an increase in valerate [46] and BCFA respectively. Phenylacetate which originates from phenylalanine is another degradation metabolite associated with a high intake of protein [29, 47] but as with BCFA no toxicity to epithelial cells has yet been reported [48]. Similar to BAA, phenylalanine is an abundant component of gut tissue [42]. This infers the possibility that a portion of phenylacetate may originate from the bacterial degradation of slough cells. The detection of a smaller amount of amino acids, sugars, secondary bile acids and other bacterial products (alcohols, polyols, amines) in faecal extracts is an expected occurrence [14, 18–19, 47].

Previous studies reported higher levels of amino acids in the extracts from the CRC patients [14, 16, 19–20] but we found an inverse trend of lower abundance of amino acids, sugars, secondary bile acids and other compounds (Table 2). This general metabolic decrease may mirror a lower “normal” bacterial activity due to the aetiology of the disease which could result in a lower concentration of metabolites in stool.

The beta-diversity analysis of the microbiota of CRC patients and healthy controls indicated that although it seemed that the faecal samples clustered by health status in the unweighted analysis, this separation of samples was less distinct when relative abundance of taxa was taken into consideration (Figure 3). This may indicate that CRC patients share a common pool of taxa, but at varying abundances. Further investigation indicated that at the family level, CRC patients harboured a greater proportion of Ruminococcaceae and a lower relative abundance of Lachnospiraceae compared to healthy controls, both of which are butyrate-producing members of the Clostridiales order. Multiple members of the Clostridiales, including *Oscillospira*, were present at higher proportions in the microbiota of CRC patients compared to healthy controls, as has been identified previously [49]. A more thorough investigation into the Clostridiales and their metabolic products may shed light on how important bacteria within this order are in colorectal cancer. The methanogenic archaea *Methanobrevibacter* was found to be present in 50% (10/20) of the CRC microbiota, compared to 10% (2/20) of the healthy controls. This taxon has previously been linked to CRC and has been considered a putative causal agent of various cancers [49], however further studies are required to elucidate the importance of this taxon in cancer, and whether gut Eukaryotes, such as fungi and protists, may play a role. A subset of 5 patients were associated to the highest levels of iso short chain fatty acids and phenylacetate. Interestingly, the bacterial taxa that were present at significantly different proportions in the proposed CRC subset (Supplementary Table 3), compared to the remaining CRC patients, have been associated with the human intestinal mucosa, inflammation, and/or an increased risk of CRC [50–51]. It was not possible to conclude further as to why those values were consistently high for those patients. Linking the microbiota and metabolomics data was attempted and although there was a weak but significant link between the two microbiota and NMR data sets, no further separation between the two groups of samples was detected (Supplementary Figure 5). Nor was a good correlation found in the heap map correlating individual metabolite with individual microbial trait (not shown).

Our sample was derived from a population-based screening programme and had each already screened positive for further investigation using FOBt and were scheduled for colonoscopy, hence our findings suggest further risk stratification using faecal metabolites might be possible within this cohort. The groups were age and sex matched, and so this cannot explain any observed differences, but we could not control for differences in lifestyle including diet which may account for our findings.

Problems of data dispersal are often encountered with chemical data originating from human samples. The ^1^H NMR spectra were characterized by heterogeneous profiles within each group (Figure 1) which showed large intra-group variability and some large outliers for specific metabolites. Nevertheless, strong evidence for differences between controls and cases for several metabolites was seen. In our cross-validated PLS-DA analysis faecal metabolites were able to discriminate between CRC and control patients with a C-statistic of 0.8, with sensitivity of 74% and specificity of 76% (classification accuracy of 75% in our sample) when a threshold of probability > 0.5 was used as a cut-off. This is less powerful than existing faecal tests, but our sample had already screened positive so we do not know what the discriminatory power would be in an unselected population. A 2014 review of faecal immunochemical tests (FIT) suggested a combined sensitivity of 79% and specificity of 94%, while faecal occult blood tests [52]. Nevertheless, our findings, if repeated in larger cohorts, suggest that faecal metabolite profiles might augment existing markers to produce a more reliable non-invasive tests and that further investigation in this area is needed.

In summary, our findings clearly demonstrate that there are significant alterations in the metabolite composition of faecal extracts from patients with CRC compared to controls. As has been reported previously, we confirmed an increase in levels of isovalerate, isobutyrate and valerate that could originate from an increase in intestinal slough cells utilisation or an increase in mucolytic activity from a subset of microbes. We also reported a decrease in levels of amino acids, sugars and various microbial products (amines, alcohols and secondary bile acids) that could be attributed to a possible generalised reduction in the metabolic activity of gut bacteria. The mechanisms underlying the observed changes are still unidentified and require further investigation.

## MATERIALS AND METHODS

### Clinical characteristics of patients

Patients referred to Addenbrookes Hospital in Cambridge, UK were enrolled after having received information about the study and given their written informed consent. Stool specimens were collected as part of a study involving the National Health Service Bowel cancer screening programme (Cambridge 2 LREC reference: 08/H0308/13). The stool samples included in the study were collected between 2009 and 2013.

All patients involved in the study had a positive Faecal occult blood test (FOBt) and were invited for a colonoscopy within the National Health Service Bowel cancer screening programme (NHSBCSP). The stool samples were collected prior to the patients starting the bowel preparation for colonoscopy and stored at −80°C immediately on arrival. Patients who had biopsy proven CRC were classified as cancers in this study. Those classified as healthy had no evidence of CRC on colonoscopy. Out of these, two sets of stool samples of patients who had colorectal cancer (CRC) and age and sex matched normal controls were analysed. The first set of samples from 40 individuals (20 CRC patients and 20 healthy controls) was analysed in 2012. A second larger set of samples (*n* = 60, 30 CRC and 30 healthy patients) was then selected and analysed in 2014. Patient demographics are shown in Table 1.

### Sample preparation

To get an adequate representation of the sample, a total of 50 g was collected from 5 different portions of the whole frozen stool block and homogenised. Samples were aliquoted in duplicates (50 mg ± 1 mg). The first aliquot was lyophilised to measure the water content which ranged from 62–89% of fresh weight for set 1 and 51–93% for set 2. To obtain the normalised metabolomics data, each metabolite value was multiplied by the ratio obtained by dividing the sample water content and the maximum water content found in the set it belongs to. The ratio factors ranged between 0.69 and 1 for set 1 and 0.59 and 1 for set 2. In each set, the values of the sample with the maximum water content were left unchanged (they were multiplied by 1) and the values of the other samples were multiplied by a factor comprised between 0.59 and 1 to compensate for their lower water content. The data were thus normalised to dry weight. The second aliquot was thawed at room temperature and prepared for ^1^H NMR spectroscopy by mixing the faecal aliquot with 600 μL NMR buffer (0.26 g NaH_2_PO_4_ and 1.41 g K_2_HPO_4_) made up in 100% D_2_O (100 ml), containing 0.1% NaN_3_ (100 mg), and 1 mM sodium 3-(Trimethylsilyl)-propionate-*d*4, (TSP) (17 mg) as a chemical shift reference. The sample was mixed, centrifuged and 500 μL was transferred into a 5-mm NMR tube for spectral acquisition. The ^1^H NMR spectra were recorded at 600 MHz on a Bruker Avance spectrometer (Bruker BioSpin GmbH, Rheinstetten, Germany) running Topspin 3.2 software and fitted with a TCI probe. Each ^1^H NMR spectrum was acquired with 2816 scans, a spectral width of 12300 Hz and an acquisition time of 2.7 s and delay time of 3 s. The “noesygppr1d” presaturation sequence was used to suppress the residual water signal with a low-power selective irradiation at the water frequency during the recycle delay. Spectra were transformed with a 0.3-Hz line broadening, manually phased, baseline corrected, and referenced by setting the TSP methyl signal to 0 ppm. Spectra were prepared for statistical analysis using the Bruker AMIX software v3.9. The “underground removal tool” of AMIX was applied to all spectra (filter width = 20 Hz) to remove the broad irregular envelope that extends from ∼0.7 to 4.5 ppm. Metabolites were identified using information found in the literature [18, 23] or on the web (Human Metabolome Database, http://www.hmdb.ca/) and by use of the 2D-NMR methods, COSY, HSQC, and HMBC. The metabolites were quantified using the NMR Suite v7.6. Profiler (Chenomx, Inc., Edmonton, Canada).

### Statistical analysis

Statistical analysis was conducted for both sets of patients concurrently. Data were described as mean, standard deviation, median and quartiles for each group (CRC and control) and the relationship between metabolites as the ratio of means between each group.

### Univariate analysis

The distribution of each metabolite concentration across participants was heavily skewed, the presence of large numbers of tied values made standard non-parametric tests impractical and large outliers in some groups made permutation tests invalid. Hence a separate Box-Cox transformation was applied to each metabolite, adding the minimum non-zero value for each metabolite to each value to enable zero values to be transformed. Visual inspection showed that the Box-Cox transformation removed skew for most metabolites well and stabilised variances across groups including outliers. Hence *t*-tests were then applied to transformed values in order to calculate the statistical significance of differences between metabolite concentrations in each group. In cases where there were large numbers of zeros the transformation was not able to remove skew, but a sensitivity analysis was conducted using Fishers exact tests comparing the proportion of non-zero metabolite values between cancer and control groups; *p*-values from Fishers exact tests in these cases were close to *p*-values from *t*-tests of transformed data supporting the validity of the *t*-tests. *P*-values from *t*-tests are reported along with adjusted *p*-values corrected for multiple testing using the procedure of Benjamini and Hochberg.

### Multivariate analysis

Scaled principal components analysis of Box-Cox transformed concentrations was used to estimate the relationships between metabolites, and to show the relationship between the first two principal components and cancer status.

To test whether metabolite concentrations predict cancer status, scaled partial least squares discriminant analysis (PLS-DA) of transformed values was then conducted. First, cross-validation was used within each set to select the number of components to use. PLS regression models using the optimal number of components were then estimated in each set and then validated in the other graphically and using C-statistics. A final model was then validated with data from both sets combined using 11-fold cross-validation, whereby the combined dataset was randomly split into 11 sets of 9 observations each, with the predictions for each group based on models estimated in the other 10. For this validation the Box-Cox transformations were re-calculated within each group before model estimation. All metabolite analysis was conducted using R statistical software version 3.5.0. Sensitivity and specificity at different thresholds and C-statistics were calculated using the pROC package. Transformations and PLS-DA model estimation were conducted using the caret package.

### 16S rRNA gene sequencing analysis

The gut microbiota composition of the first cohort (20 CRC versus 20 age and sex matched healthy patients) was assessed using 454 pyrosequencing. The FastDNA SPIN Kit for Soil (MP Biomedicals, UK) was used following the manufacturer's instructions, with an additional bead-beating step, to extract the microbial DNA from the collected faecal samples. The quality and yield of the DNA was assessed using gel electrophoresis, and the NanoDrop ND-1000 UV/vis spectrophotometer (NanoDrop Technologies, Inc., USA), respectively. The DNA was sent to the Animal Health and Veterinary Laboratories Agency (UK), where the V4 and V5 regions of the 16S rRNA genes were amplified using the U515F (5′-GTGYCAGCMGCCGCGGTA) and U927R (5′-CCCGYCAATTCMTTTRAGT) primers, prior to the amplicons being subjected to 454 pyrosequencing [53]. Analysis of the sequencing reads was performed using Quantitative Insights Into Microbial Ecology (QIIME) 1.9.1 software and RDP classifier (version 2.10) 16S rRNA gene sequence database [54]. All sequences were filtered to meet the following criteria: read length between 200 and 1,000 bp; maximum of 6 ambiguous bases; minimum average quality score of 25 within a 50 bp window; and exact match to primer sequences. ChimeraSlayer was used to filter the trimmed reads for chimeric sequences, RDP classifier enabled microbial taxonomy assignment with a confidence value threshold of 50%, and the trimmed reads clustered into operational taxonomic units at 97% identity level.

## SUPPLEMENTARY MATERIALS FIGURES AND TABLES






