# Correction: Metabolite quantification of faecal extracts from colorectal cancer patients and healthy controls

**DOI:** 10.18632/oncotarget.26766

**Published:** 2019-02-26

**Authors:** Gwénaëlle Le Gall, Kiran Guttula, Lee Kellingray, Adrian J. Tett, Rogier ten Hoopen, E. Kate Kemsley, George M. Savva, Ashraf Ibrahim, Arjan Narbad

**Affiliations:** ^1^ Quadram Institute Bioscience, Norwich Research Park, Norwich, UK; ^2^ Division of Molecular Histopathology, Department of Pathology, University of Cambridge, Cambridge, UK; ^3^ Centre for Integrative Biology, University of Trento, Trento, Italy

**This article has been corrected:** The correct author name is given below:

**E. Kate Kemsley**

Original article: Oncotarget. 2018; 9:33278-33289. https://doi.org/10.18632/oncotarget.26022

