# Supplementary material for: Metabolite quantification of faecal extracts from colorectal cancer patients and healthy controls
Source: Oncotarget. 2018 Sep 7;9(70):33278–89. doi: 10.18632/oncotarget.26022 (PMC6161785; doi:10.18632/oncotarget.26022)
Supplement: Supplementary file 1 [file oncotarget-09-33278-s001.pdf]

Metabolite quantification of faecal extracts from colorectal cancer patients and healthy controls

SUPPLEMENTARY MATERIALS

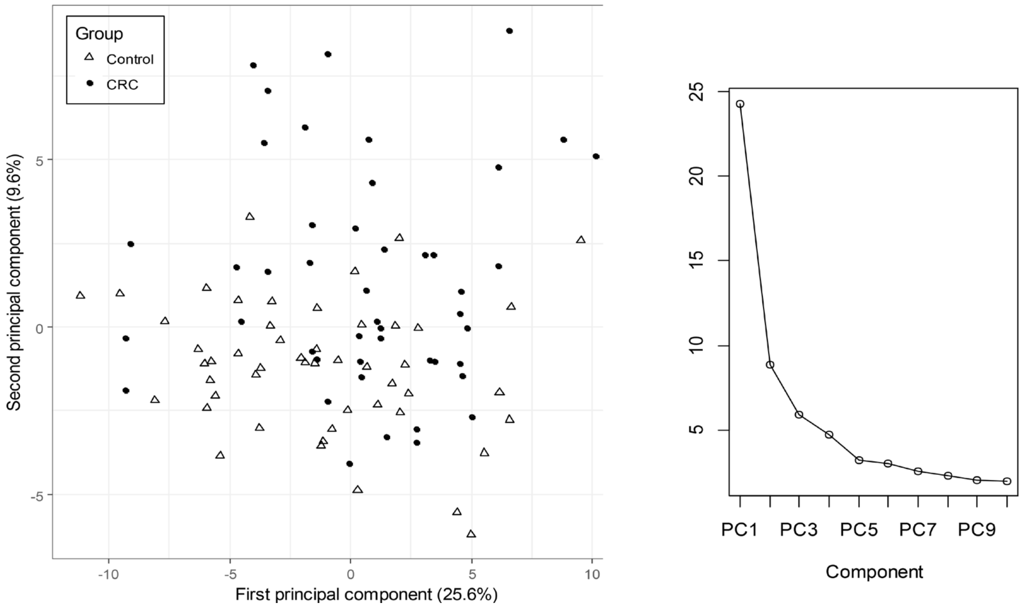

Supplementary Figure 1: PCA score plot (left panel) and associated scree plot showing the variance explained (out of 99) by each component, for PCA applied the NMR data of both CRC patients and controls. Cancer status appears related to both components 1 and 2, the scree plot suggests no clear cut-off for the number of components needed to account for variation in the data.

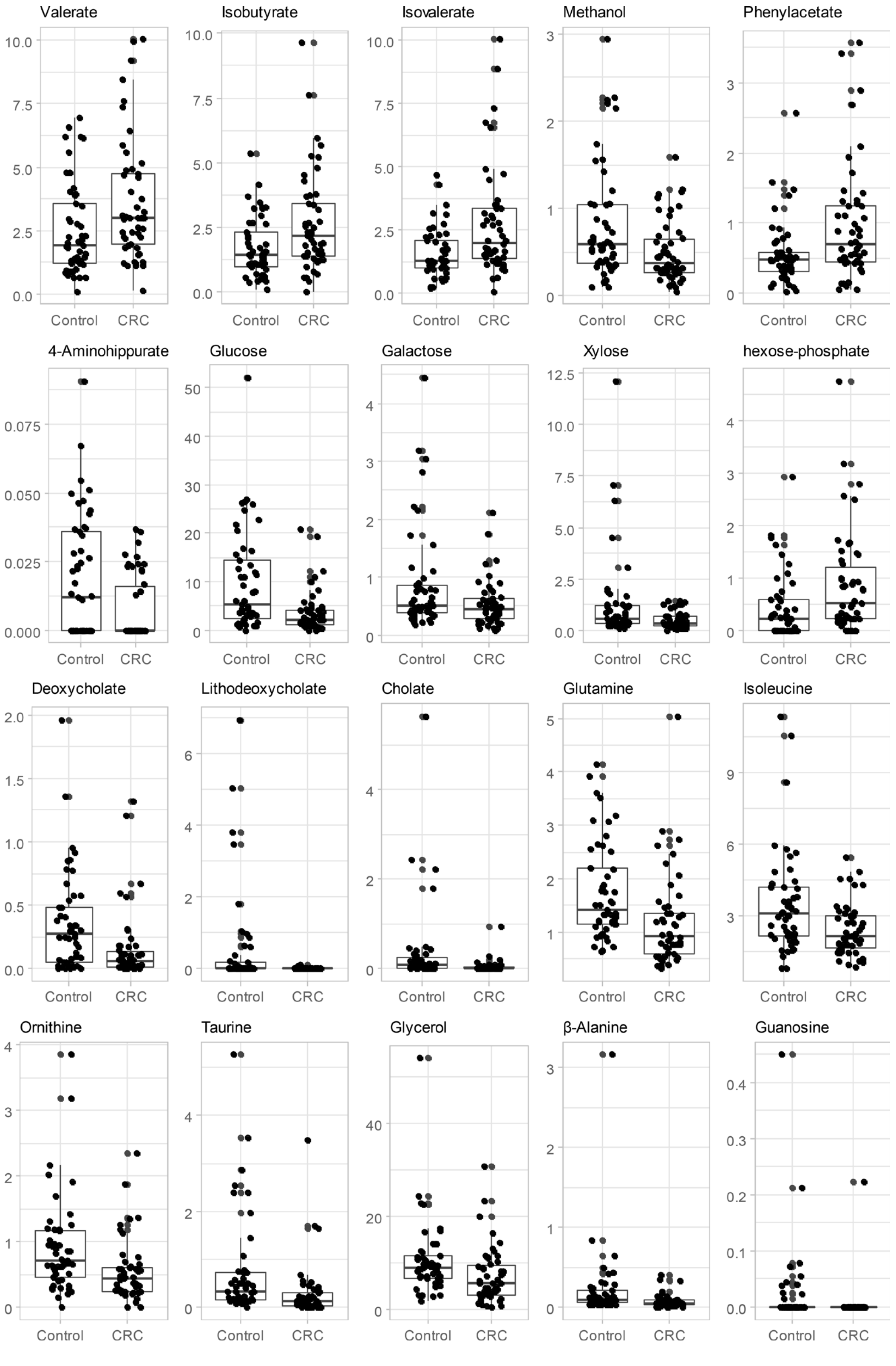

Supplementary Figure 2: Concentrations of metabolite values (mmol/kg) among cancer patients and controls, where the differences are statistically significantly different between groups (at FDR adjusted *p*-value < 0.05).

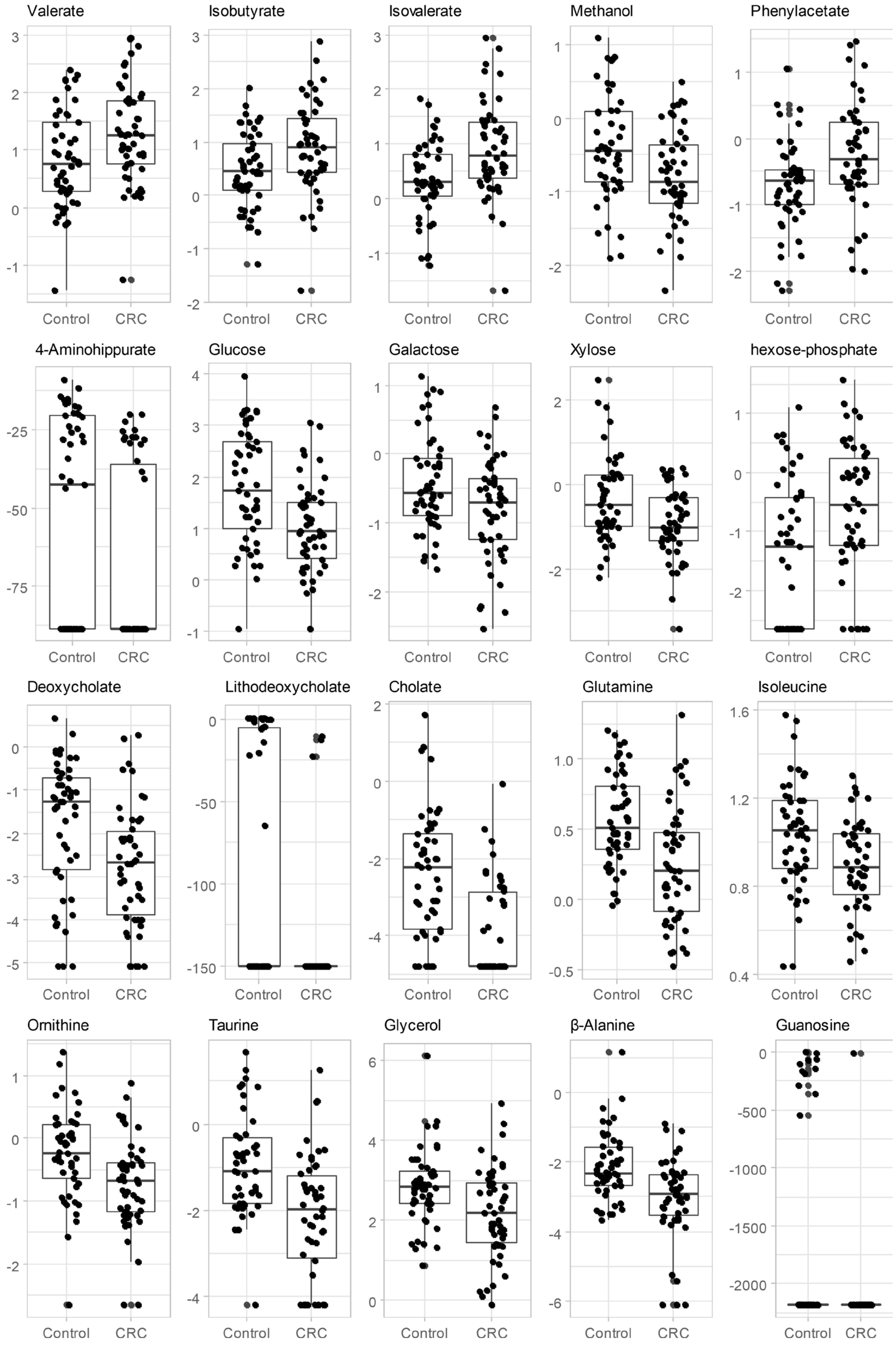

Supplementary Figure 3: Box-Cox transformed metabolite concentrations among cancer and control patients. where the differences are statistically significantly different between groups (at FDR adjusted *p*-value < 0.05).

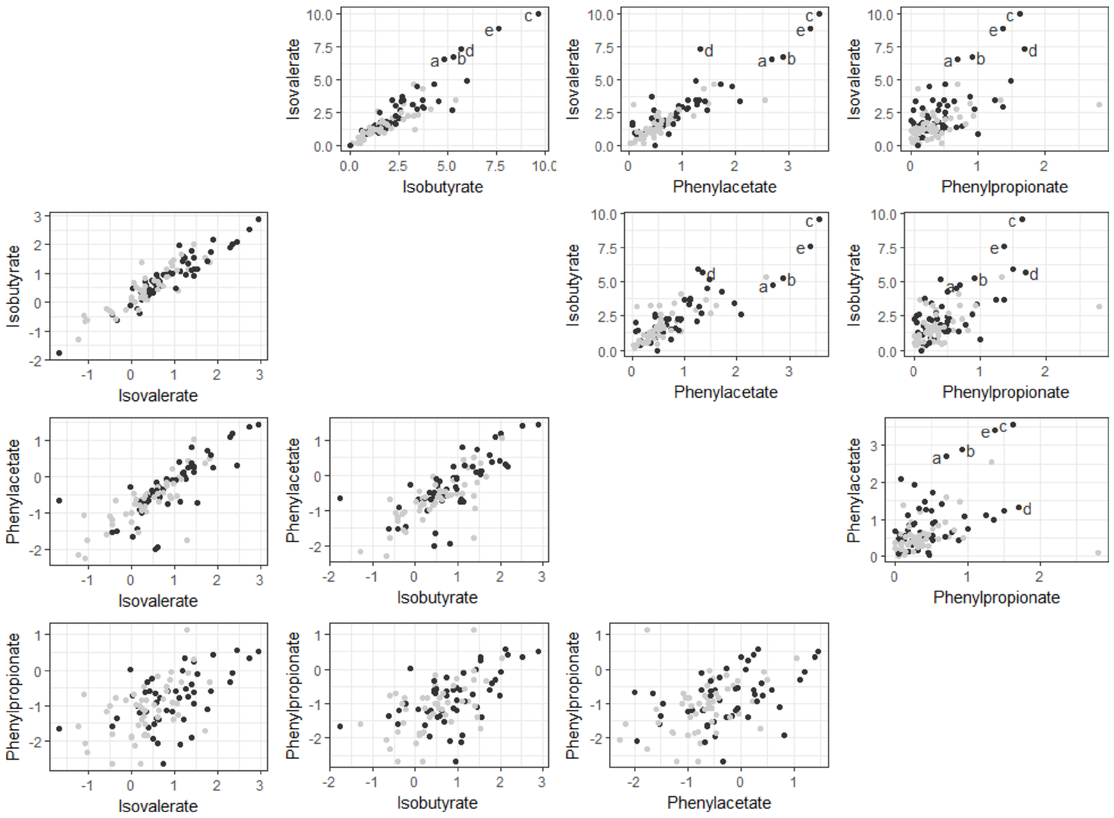

Supplementary Figure 4: pairwise correlations between concentrations of isovalerate, isobutryrate, phenylacetate and phenylproprionate. Upper triangle shows raw concentrations (mmol/kg), lower triangle shows Box-Cox transformed values. Five cancer patients responsible for high values in each metabolite are labelled in the raw data.

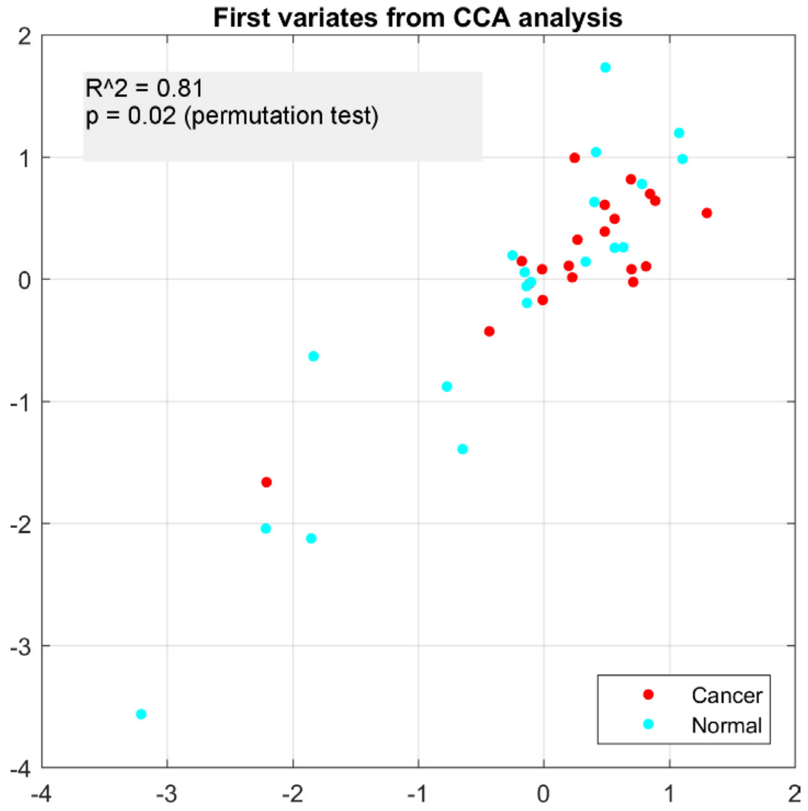

Supplementary Figure 5: First two variates by canonical correlation analysis (CCA) of the nmr and microbiota data from cohort 1. Both datasets were passed to the CCA as principal component scores, in each case accounting for 95% of the variance in the respective original datasets. The *p*-value was obtained by permutation resampling (of observation order). For information, the plot symbols indicate the cancer and non-cancer groups. This indicates that whilst the datasets are correlated to an extent, the common information is not primarily associated with the difference between the two groups.

Supplementary Table 1: The distribution of metabolite concentrations (mmol/kg) among patients with CRC and controls, along with the ratio of mean values, *p*-values on *t*-statistics and FDR *p*-values. See Supplementary_Table_1

Supplementary Table 2: Ratio of means and statistical significance of differences between groups as per Supplementary Table 1 for combined data, with individual ratios and *p*-values calculated among set 1 (*N* = 40) and set 2 (*N* = 59). See Supplementary_Table_2

Supplementary Table 3: Statistically significant taxa that differ between colorectal cancer subset (K13, K15, K21, and K37 (present in a minimum of 2 samples)), and remaining colorectal cancer patients

Microbial taxa	CRC subset	CRC others	*P* value
o_Lactobacillales	0.03 ± 0.03	<0.01 ± 0.01	0.021
f_Barnesiellaceae	0.68 ± 0.80	0.16 ± 0.19	0.021
g_Odoribacter	0.23 ± 0.21	0.09 ± 0.09	0.047
g_Enterococcus	2.21 ± 4.02	<0.01 ± 0.02	0.027
g_Megasphaera	0.36 ± 0.01	<0.01 ± 0.01	0.009
g_Mogibacterium	1.10 ± 1.49	0.11 ± 0.25	0.014

Preceding letter indicates taxonomic level: o = order; f = family; g = genus. Values shown are mean ± SD.
