# Supplementary material for: Metabolite quantification of faecal extracts from colorectal cancer patients and healthy controls
Source: Oncotarget. 2018 Sep 7;9(70):33278–89. doi: 10.18632/oncotarget.26022 (PMC6161785; doi:10.18632/oncotarget.26022)
Supplement: Supplementary file 2 [file oncotarget-09-33278-s002.docx]

**Supplementary Table 1:** The distribution of metabolite concentrations (mmol/kg) among patients with CRC and controls, along with the ratio of mean values. P-values are calculated based on t-statistics calculated using Box-Cox transformations of the raw concentrations. FDR p-values are calculated based on 94 hypothesis tests being conducted using the Benjamini and Hochberg procedure.

|  | CRC patients | | | | | Controls | | | | |  | | | |
| --- | --- | --- | --- | --- | --- | --- | --- | --- | --- | --- | --- | --- | --- | --- |
| Metabolite | mean | sd | P25 | Median | P75 | mean | sd | P25 | Median | P75 | Ratio of means | t | p-value | FDR corrected p-value |
| Butyrate | 14.46 | 11.27 | 5.84 | 13.40 | 19.70 | 16.41 | 13.55 | 6.42 | 13.14 | 21.12 | 0.88 | -0.53 | 0.599 | 0.663 |
| Acetate | 58.21 | 31.59 | 36.95 | 56.17 | 71.68 | 61.32 | 33.97 | 36.72 | 49.23 | 78.18 | 0.95 | -0.44 | 0.659 | 0.712 |
| Propionate | 17.12 | 9.67 | 10.52 | 14.87 | 22.36 | 20.12 | 16.67 | 9.99 | 17.33 | 22.41 | 0.85 | -0.77 | 0.446 | 0.582 |
| Valerate | 3.59 | 2.41 | 1.94 | 2.99 | 4.74 | 2.53 | 1.82 | 1.22 | 1.91 | 3.55 | 1.42 | 2.62 | 0.010 | 0.050 |
| Isobutyrate | 2.62 | 1.90 | 1.40 | 2.17 | 3.42 | 1.70 | 1.14 | 0.98 | 1.43 | 2.31 | 1.54 | 2.74 | 0.007 | 0.041 |
| Isovalerate | 2.73 | 2.10 | 1.37 | 2.00 | 3.36 | 1.56 | 1.01 | 0.98 | 1.27 | 2.07 | 1.75 | 3.59 | 0.001 | 0.007 |
| 2-methylbutyate | 1.01 | 0.64 | 0.58 | 0.86 | 1.31 | 0.79 | 0.53 | 0.41 | 0.64 | 1.10 | 1.27 | 2.05 | 0.043 | 0.118 |
| Lactate | 0.39 | 0.28 | 0.21 | 0.36 | 0.55 | 0.76 | 0.89 | 0.30 | 0.45 | 0.88 | 0.51 | -2.14 | 0.035 | 0.103 |
| Ethanol | 0.65 | 0.47 | 0.38 | 0.46 | 0.74 | 0.92 | 1.05 | 0.33 | 0.53 | 1.12 | 0.70 | -0.70 | 0.486 | 0.611 |
| Methanol | 0.50 | 0.35 | 0.27 | 0.38 | 0.65 | 0.81 | 0.66 | 0.37 | 0.59 | 1.05 | 0.62 | -2.77 | 0.007 | 0.040 |
| Formate | 1.19 | 1.02 | 0.36 | 0.61 | 1.98 | 1.34 | 1.22 | 0.41 | 0.67 | 2.35 | 0.89 | -0.29 | 0.769 | 0.804 |
| Phenylpropionate | 0.51 | 0.44 | 0.19 | 0.41 | 0.67 | 0.38 | 0.45 | 0.12 | 0.28 | 0.45 | 1.34 | 1.85 | 0.068 | 0.145 |
| Phenylacetate | 0.96 | 0.81 | 0.45 | 0.70 | 1.24 | 0.55 | 0.46 | 0.30 | 0.48 | 0.59 | 1.73 | 3.01 | 0.003 | 0.021 |
| 3-hydroxyphenylpropionate | 0.03 | 0.12 | 0.00 | 0.00 | 0.00 | 0.06 | 0.12 | 0.00 | 0.00 | 0.07 | 0.57 | -2.09 | 0.039 | 0.111 |
| 3-Hydroxyphenylacetate | 0.06 | 0.09 | 0.00 | 0.00 | 0.10 | 0.05 | 0.07 | 0.00 | 0.01 | 0.08 | 1.24 | 0.01 | 0.996 | 0.996 |
| 3-Hydroxyphenyl compound 2 | 0.15 | 0.36 | 0.00 | 0.00 | 0.00 | 0.03 | 0.09 | 0.00 | 0.00 | 0.00 | 5.18 | 1.56 | 0.123 | 0.231 |
| 4-Hydroxyphenylacetate | 0.05 | 0.09 | 0.00 | 0.02 | 0.06 | 0.10 | 0.27 | 0.00 | 0.03 | 0.07 | 0.47 | -0.30 | 0.767 | 0.804 |
| 4-Hydroxybenzoate | 0.01 | 0.02 | 0.00 | 0.00 | 0.02 | 0.01 | 0.03 | 0.00 | 0.00 | 0.00 | 0.64 | 0.87 | 0.388 | 0.521 |
| 4-Aminohippurate | 0.01 | 0.01 | 0.00 | 0.00 | 0.02 | 0.02 | 0.02 | 0.00 | 0.01 | 0.04 | 0.39 | -2.61 | 0.011 | 0.050 |
| 6-Hydroxynicotinate | 0.04 | 0.11 | 0.00 | 0.00 | 0.00 | 0.06 | 0.15 | 0.00 | 0.00 | 0.00 | 0.60 | -0.59 | 0.557 | 0.623 |
| Benzoate | 0.01 | 0.02 | 0.00 | 0.00 | 0.02 | 0.02 | 0.02 | 0.00 | 0.02 | 0.03 | 0.70 | -1.43 | 0.156 | 0.272 |
| Phenol | 0.04 | 0.16 | 0.00 | 0.00 | 0.00 | 0.03 | 0.07 | 0.00 | 0.00 | 0.00 | 1.27 | -0.63 | 0.530 | 0.623 |
| p-Cresol | 0.07 | 0.09 | 0.00 | 0.04 | 0.11 | 0.03 | 0.05 | 0.00 | 0.00 | 0.04 | 2.34 | 2.31 | 0.023 | 0.074 |
| Ferulate | 0.00 | 0.01 | 0.00 | 0.00 | 0.00 | 0.01 | 0.03 | 0.00 | 0.00 | 0.00 | 0.17 | -2.47 | 0.016 | 0.064 |
| Methylamine | 0.20 | 0.17 | 0.10 | 0.18 | 0.23 | 0.19 | 0.14 | 0.09 | 0.15 | 0.24 | 1.09 | 0.64 | 0.524 | 0.623 |
| Dimethylamine | 0.07 | 0.05 | 0.03 | 0.06 | 0.08 | 0.05 | 0.03 | 0.04 | 0.05 | 0.08 | 1.26 | 1.24 | 0.218 | 0.320 |
| Trimethylamine | 0.13 | 0.13 | 0.05 | 0.09 | 0.16 | 0.14 | 0.22 | 0.02 | 0.08 | 0.15 | 0.89 | 0.67 | 0.505 | 0.623 |
| Cadaverine | 1.42 | 2.72 | 0.22 | 0.64 | 1.36 | 1.05 | 1.15 | 0.00 | 0.73 | 1.83 | 1.36 | 0.27 | 0.788 | 0.814 |
| Tyramine | 0.06 | 0.15 | 0.00 | 0.00 | 0.06 | 0.15 | 0.41 | 0.00 | 0.00 | 0.14 | 0.41 | -0.33 | 0.740 | 0.791 |
| Putrescine | 0.11 | 0.28 | 0.00 | 0.00 | 0.00 | 0.55 | 1.21 | 0.00 | 0.00 | 0.38 | 0.20 | -2.14 | 0.035 | 0.103 |
| N-Acetylputrescine | 0.01 | 0.06 | 0.00 | 0.00 | 0.00 | 0.11 | 0.31 | 0.00 | 0.00 | 0.00 | 0.11 | -2.19 | 0.031 | 0.098 |
| Glucose | 3.69 | 4.35 | 1.12 | 2.18 | 4.14 | 9.80 | 10.35 | 2.30 | 5.25 | 14.35 | 0.38 | -3.91 | 0.000 | 0.003 |
| Galactose | 0.53 | 0.40 | 0.28 | 0.46 | 0.63 | 0.87 | 0.90 | 0.38 | 0.52 | 0.86 | 0.61 | -2.66 | 0.009 | 0.048 |
| Arabinose | 0.17 | 0.32 | 0.00 | 0.08 | 0.16 | 0.46 | 1.47 | 0.00 | 0.22 | 0.38 | 0.37 | -1.98 | 0.050 | 0.127 |
| Xylose | 0.50 | 0.40 | 0.23 | 0.33 | 0.70 | 1.31 | 2.12 | 0.33 | 0.59 | 1.22 | 0.38 | -3.31 | 0.001 | 0.012 |
| Ribose | 2.97 | 1.79 | 1.44 | 2.78 | 3.81 | 4.44 | 3.44 | 2.55 | 3.76 | 5.52 | 0.67 | -2.39 | 0.019 | 0.071 |
| Fucose | 0.13 | 0.16 | 0.00 | 0.06 | 0.21 | 0.24 | 0.41 | 0.00 | 0.07 | 0.28 | 0.54 | -0.61 | 0.543 | 0.623 |
| hexose-phosphate | 0.85 | 0.96 | 0.22 | 0.51 | 1.19 | 0.42 | 0.63 | 0.00 | 0.21 | 0.59 | 2.02 | 3.37 | 0.001 | 0.012 |
| Myo-Inositol | 0.11 | 0.18 | 0.00 | 0.00 | 0.20 | 0.24 | 0.32 | 0.00 | 0.10 | 0.34 | 0.48 | -1.96 | 0.053 | 0.127 |
| Deoxycholate | 0.16 | 0.27 | 0.01 | 0.06 | 0.14 | 0.36 | 0.39 | 0.05 | 0.27 | 0.48 | 0.44 | -3.28 | 0.001 | 0.012 |
| Lithodeoxycholate | 0.00 | 0.02 | 0.00 | 0.00 | 0.00 | 0.54 | 1.40 | 0.00 | 0.00 | 0.17 | 0.01 | -4.02 | 0.000 | 0.003 |
| Cholate | 0.05 | 0.14 | 0.00 | 0.00 | 0.05 | 0.36 | 0.93 | 0.01 | 0.10 | 0.25 | 0.13 | -5.06 | 0.000 | 0.000 |
| Alanine | 0.92 | 0.85 | 0.41 | 0.65 | 1.13 | 1.30 | 1.31 | 0.56 | 0.84 | 1.22 | 0.70 | -1.85 | 0.068 | 0.145 |
| Asparagine | 0.20 | 0.39 | 0.00 | 0.08 | 0.18 | 0.32 | 0.44 | 0.00 | 0.16 | 0.42 | 0.64 | -1.29 | 0.199 | 0.306 |
| Aspartate | 1.14 | 1.37 | 0.25 | 0.63 | 1.28 | 1.36 | 1.22 | 0.58 | 1.09 | 1.71 | 0.83 | -1.88 | 0.063 | 0.145 |
| Citrulline | 1.22 | 0.90 | 0.67 | 1.07 | 1.46 | 1.36 | 0.96 | 0.75 | 1.18 | 1.86 | 0.90 | -0.60 | 0.550 | 0.623 |
| Glutamate | 6.67 | 3.05 | 4.33 | 6.13 | 8.88 | 7.97 | 4.82 | 4.73 | 7.31 | 9.46 | 0.84 | -1.34 | 0.184 | 0.299 |
| Glutamine | 1.18 | 0.86 | 0.60 | 0.92 | 1.36 | 1.76 | 0.91 | 1.15 | 1.41 | 2.21 | 0.67 | -4.24 | 0.000 | 0.002 |
| Glycine | 2.08 | 1.49 | 1.30 | 1.55 | 2.59 | 3.09 | 2.24 | 1.52 | 2.42 | 4.20 | 0.67 | -2.32 | 0.022 | 0.074 |
| Histidine | 0.39 | 0.59 | 0.08 | 0.18 | 0.46 | 0.49 | 0.40 | 0.21 | 0.38 | 0.64 | 0.78 | -2.45 | 0.016 | 0.064 |
| Urocanate | 0.03 | 0.04 | 0.00 | 0.02 | 0.04 | 0.05 | 0.06 | 0.00 | 0.04 | 0.06 | 0.72 | -1.28 | 0.204 | 0.306 |
| Isoleucine | 2.45 | 1.08 | 1.67 | 2.17 | 3.02 | 3.52 | 2.15 | 2.16 | 3.13 | 4.19 | 0.70 | -3.01 | 0.003 | 0.021 |
| Leucine | 4.06 | 2.54 | 2.37 | 3.20 | 4.65 | 4.94 | 3.02 | 3.40 | 4.08 | 5.68 | 0.82 | -1.86 | 0.066 | 0.145 |
| Lysine | 3.60 | 2.17 | 2.08 | 3.27 | 4.42 | 4.43 | 2.52 | 2.52 | 4.19 | 5.27 | 0.81 | -1.81 | 0.073 | 0.152 |
| Glutarate | 0.39 | 0.38 | 0.05 | 0.25 | 0.70 | 0.37 | 0.40 | 0.00 | 0.28 | 0.56 | 1.05 | 0.62 | 0.538 | 0.623 |
| 5-Aminovalerate | 0.33 | 0.25 | 0.16 | 0.26 | 0.45 | 0.57 | 0.86 | 0.17 | 0.28 | 0.48 | 0.59 | -1.28 | 0.205 | 0.306 |
| 2-Piperidinone | 0.02 | 0.08 | 0.00 | 0.00 | 0.00 | 0.08 | 0.28 | 0.00 | 0.00 | 0.00 | 0.22 | -1.15 | 0.253 | 0.366 |
| Methionine | 0.95 | 0.51 | 0.55 | 0.85 | 1.15 | 1.21 | 0.75 | 0.76 | 1.15 | 1.41 | 0.79 | -1.88 | 0.063 | 0.145 |
| Ornithine | 0.54 | 0.46 | 0.24 | 0.44 | 0.60 | 0.91 | 0.73 | 0.46 | 0.71 | 1.16 | 0.59 | -3.33 | 0.001 | 0.012 |
| Phenylalanine | 1.72 | 1.23 | 0.89 | 1.38 | 1.87 | 2.12 | 1.36 | 1.39 | 1.77 | 2.44 | 0.81 | -1.97 | 0.051 | 0.127 |
| Proline | 1.21 | 0.80 | 0.68 | 1.12 | 1.46 | 1.43 | 0.95 | 0.89 | 1.26 | 1.65 | 0.85 | -1.13 | 0.262 | 0.373 |
| Serine | 1.33 | 0.89 | 0.64 | 1.12 | 1.89 | 1.84 | 1.38 | 1.02 | 1.51 | 1.95 | 0.72 | -2.52 | 0.014 | 0.061 |
| Taurine | 0.29 | 0.57 | 0.03 | 0.12 | 0.29 | 0.73 | 1.05 | 0.14 | 0.33 | 0.71 | 0.39 | -4.59 | 0.000 | 0.001 |
| Threonine | 2.05 | 1.24 | 1.21 | 1.60 | 2.51 | 2.55 | 1.36 | 1.47 | 2.27 | 3.28 | 0.80 | -2.04 | 0.044 | 0.119 |
| Tryptophan | 0.30 | 0.25 | 0.13 | 0.22 | 0.32 | 0.39 | 0.23 | 0.22 | 0.36 | 0.47 | 0.76 | -2.46 | 0.016 | 0.064 |
| Tyrosine | 1.74 | 0.93 | 1.03 | 1.57 | 2.09 | 2.28 | 1.34 | 1.47 | 2.01 | 2.78 | 0.76 | -2.34 | 0.021 | 0.074 |
| Valine | 3.81 | 2.61 | 2.07 | 2.87 | 4.63 | 4.62 | 3.05 | 2.74 | 4.10 | 5.08 | 0.82 | -1.66 | 0.100 | 0.195 |
| Methylsuccinate | 0.11 | 0.12 | 0.00 | 0.08 | 0.18 | 0.09 | 0.10 | 0.01 | 0.07 | 0.11 | 1.21 | 0.46 | 0.645 | 0.705 |
| 3-Methyl-2-oxovalerate | 2.12 | 3.35 | 0.00 | 1.03 | 2.91 | 2.70 | 3.30 | 0.00 | 1.88 | 3.15 | 0.79 | -1.42 | 0.160 | 0.274 |
| N6-Acetyllysine | 0.17 | 0.56 | 0.00 | 0.00 | 0.00 | 0.36 | 0.77 | 0.00 | 0.00 | 0.27 | 0.48 | -1.71 | 0.091 | 0.182 |
| methylamino acid | 0.25 | 0.17 | 0.14 | 0.19 | 0.30 | 0.35 | 0.26 | 0.20 | 0.30 | 0.40 | 0.71 | -2.35 | 0.021 | 0.074 |
| Succinate | 2.19 | 12.36 | 0.13 | 0.20 | 0.47 | 1.64 | 4.92 | 0.12 | 0.22 | 0.82 | 1.34 | -1.38 | 0.170 | 0.280 |
| Pyruvate | 3.44 | 2.20 | 1.72 | 2.75 | 4.57 | 4.13 | 2.77 | 2.60 | 3.42 | 4.90 | 0.83 | -1.44 | 0.153 | 0.272 |
| Fumarate | 0.12 | 0.13 | 0.03 | 0.08 | 0.13 | 0.12 | 0.10 | 0.05 | 0.08 | 0.17 | 0.98 | -0.80 | 0.426 | 0.563 |
| Malate | 0.20 | 0.35 | 0.00 | 0.00 | 0.32 | 0.33 | 0.48 | 0.00 | 0.00 | 0.48 | 0.60 | -1.40 | 0.165 | 0.278 |
| 1,3-Dihydroxyacetone | 0.03 | 0.02 | 0.01 | 0.03 | 0.04 | 0.04 | 0.04 | 0.01 | 0.03 | 0.04 | 0.88 | -0.01 | 0.993 | 0.996 |
| Malonate | 1.06 | 2.13 | 0.32 | 0.58 | 0.82 | 1.31 | 1.92 | 0.43 | 0.67 | 1.09 | 0.81 | -1.65 | 0.102 | 0.196 |
| 3-Hydroxybutyrate | 0.11 | 0.40 | 0.00 | 0.03 | 0.08 | 0.06 | 0.08 | 0.00 | 0.03 | 0.09 | 1.88 | 0.60 | 0.548 | 0.623 |
| Acetone | 0.13 | 0.30 | 0.00 | 0.00 | 0.09 | 0.08 | 0.18 | 0.00 | 0.00 | 0.00 | 1.63 | 0.70 | 0.488 | 0.611 |
| Glycerol | 7.18 | 6.01 | 3.13 | 5.65 | 9.54 | 10.53 | 8.13 | 6.76 | 9.04 | 11.56 | 0.68 | -3.20 | 0.002 | 0.015 |
| Isopropanol | 0.09 | 0.07 | 0.04 | 0.07 | 0.11 | 0.08 | 0.17 | 0.03 | 0.05 | 0.08 | 1.07 | 2.00 | 0.048 | 0.126 |
| Propylene glycol | 0.06 | 0.13 | 0.00 | 0.01 | 0.07 | 0.05 | 0.12 | 0.00 | 0.00 | 0.04 | 1.21 | 1.08 | 0.281 | 0.395 |
| Choline | 0.10 | 0.08 | 0.05 | 0.08 | 0.12 | 0.14 | 0.17 | 0.06 | 0.09 | 0.14 | 0.67 | -1.47 | 0.145 | 0.266 |
| Dimethylglycine | 0.03 | 0.03 | 0.01 | 0.01 | 0.03 | 0.04 | 0.06 | 0.01 | 0.02 | 0.05 | 0.61 | -0.59 | 0.557 | 0.623 |
| Hypoxanthine | 1.22 | 0.63 | 0.81 | 1.10 | 1.50 | 1.47 | 0.99 | 0.91 | 1.28 | 1.75 | 0.83 | -1.00 | 0.322 | 0.438 |
| Uracil | 1.71 | 0.83 | 1.22 | 1.51 | 2.17 | 2.15 | 1.41 | 1.13 | 1.80 | 2.67 | 0.79 | -1.43 | 0.156 | 0.272 |
| β-Alanine | 0.08 | 0.08 | 0.03 | 0.05 | 0.09 | 0.22 | 0.46 | 0.07 | 0.10 | 0.20 | 0.35 | -4.13 | 0.000 | 0.002 |
| Uridine | 0.02 | 0.05 | 0.00 | 0.00 | 0.03 | 0.05 | 0.10 | 0.00 | 0.00 | 0.10 | 0.50 | 0.03 | 0.973 | 0.994 |
| Xanthine | 1.93 | 1.01 | 1.12 | 1.72 | 2.61 | 2.37 | 1.57 | 1.38 | 2.17 | 2.89 | 0.81 | -1.30 | 0.198 | 0.306 |
| Cytidine | 0.01 | 0.01 | 0.00 | 0.00 | 0.00 | 0.01 | 0.03 | 0.00 | 0.00 | 0.02 | 0.44 | -1.28 | 0.204 | 0.306 |
| Guanosine | 0.00 | 0.03 | 0.00 | 0.00 | 0.00 | 0.02 | 0.07 | 0.00 | 0.00 | 0.00 | 0.21 | -3.16 | 0.002 | 0.018 |
| Inosine | 0.02 | 0.05 | 0.00 | 0.00 | 0.03 | 0.03 | 0.06 | 0.00 | 0.00 | 0.03 | 0.80 | 0.70 | 0.486 | 0.611 |
| Niacinamide | 0.01 | 0.02 | 0.00 | 0.00 | 0.00 | 0.01 | 0.03 | 0.00 | 0.00 | 0.00 | 1.03 | 1.00 | 0.321 | 0.438 |
| Nicotinate | 0.16 | 0.10 | 0.08 | 0.14 | 0.22 | 0.20 | 0.14 | 0.12 | 0.16 | 0.22 | 0.79 | -1.72 | 0.088 | 0.180 |
