# Supplementary material for: Metabolite quantification of faecal extracts from colorectal cancer patients and healthy controls
Source: Oncotarget. 2018 Sep 7;9(70):33278–89. doi: 10.18632/oncotarget.26022 (PMC6161785; doi:10.18632/oncotarget.26022)
Supplement: Supplementary file 3 [file oncotarget-09-33278-s003.docx]

**Supplementary Table 2**. Ratio of means and statistical significance of differences between groups as per supplementary table 1 for combined data, with individual ratios and p-values calculated among set 1 (N=40) and set 2 (N=59). P-values for interactions test the null hypothesis of no difference between sets in the association between cancer status and metabolite values.

|  | Combined data | | | | Set 1 | | Set 2 | | Interaction |
| --- | --- | --- | --- | --- | --- | --- | --- | --- | --- |
|  | Ratio of means | t | p-value  (t-test) | Adjusted p-value (FDR) | Ratio of means | p-value (t-test) | Ratio of means | p-value (t-test) | P-value for interaction |
| Butyrate | 0.88 | -0.53 | 0.599 | 0.663 | 0.77 | 0.359 | 0.96 | 0.998 | 0.523 |
| Acetate | 0.95 | -0.44 | 0.659 | 0.712 | 0.88 | 0.450 | 0.99 | 0.967 | 0.637 |
| Propionate | 0.85 | -0.77 | 0.446 | 0.582 | 0.78 | 0.233 | 0.91 | 0.932 | 0.441 |
| Valerate | 1.42 | 2.62 | 0.010 | 0.050 | 1.30 | 0.293 | 1.51 | 0.016 | 0.456 |
| Isobutyrate | 1.54 | 2.74 | 0.007 | 0.041 | 1.54 | 0.149 | 1.54 | 0.022 | 0.855 |
| Isovalerate | 1.75 | 3.59 | 0.001 | 0.007 | 1.62 | 0.100 | 1.85 | 0.001 | 0.764 |
| 2-methylbutyate | 1.27 | 2.05 | 0.043 | 0.118 | 1.12 | 0.496 | 1.39 | 0.024 | 0.541 |
| Lactate | 0.51 | -2.14 | 0.035 | 0.103 | 0.61 | 0.934 | 0.46 | 0.001 | 0.092 |
| Ethanol | 0.70 | -0.70 | 0.486 | 0.611 | 0.63 | 0.947 | 0.80 | 0.413 | 0.621 |
| Methanol | 0.62 | -2.77 | 0.007 | 0.040 | 0.69 | 0.249 | 0.58 | 0.003 | 0.628 |
| Formate | 0.89 | -0.29 | 0.769 | 0.804 | 0.86 | 0.116 | 1.05 | 0.667 | 0.166 |
| Phenylpropionate | 1.34 | 1.85 | 0.068 | 0.145 | 1.14 | 0.381 | 1.54 | 0.087 | 0.856 |
| Phenylacetate | 1.73 | 3.01 | 0.003 | 0.021 | 1.63 | 0.183 | 1.80 | 0.003 | 0.790 |
| 3-hydroxyphenylpropionate | 0.57 | -2.09 | 0.039 | 0.111 | 0.46 | 0.104 | 0.65 | 0.200 | 0.567 |
| 3-Hydroxyphenylacetate | 1.24 | 0.01 | 0.996 | 0.996 | 1.20 | 0.558 | 1.28 | 0.676 | 0.481 |
| 3-Hydroxyphenyl compound 2 | 5.18 | 1.56 | 0.123 | 0.231 | 3.36 | 0.360 | 10.00 | 0.168 | 0.869 |
| 4-Hydroxyphenylacetate | 0.47 | -0.30 | 0.767 | 0.804 | 0.88 | 0.920 | 0.27 | 0.679 | 0.726 |
| 4-Hydroxybenzoate | 0.64 | 0.87 | 0.388 | 0.521 | 0.52 | 0.920 | 3.62 | 0.102 | 0.504 |
| 4-Aminohippurate | 0.39 | -2.61 | 0.011 | 0.050 | 0.36 | 0.001 | 0.44 | 0.463 | 0.035 |
| 6-Hydroxynicotinate | 0.60 | -0.59 | 0.557 | 0.623 | 0.70 | 0.444 | 0.49 | 0.944 | 0.549 |
| Benzoate | 0.70 | -1.43 | 0.156 | 0.272 | 0.72 | 0.075 | 0.69 | 0.714 | 0.246 |
| Phenol | 1.27 | -0.63 | 0.530 | 0.623 | 2.24 | 0.693 | 0.73 | 0.624 | 0.974 |
| p-Cresol | 2.34 | 2.31 | 0.023 | 0.074 | 1.02 | 0.696 | 5.40 | 0.001 | 0.015 |
| Ferulate | 0.17 | -2.47 | 0.016 | 0.064 | NA | 0.022 | 0.50 | 0.316 | 0.124 |
| Methylamine | 1.09 | 0.64 | 0.524 | 0.623 | 0.79 | 0.300 | 1.35 | 0.131 | 0.081 |
| Dimethylamine | 1.26 | 1.24 | 0.218 | 0.320 | 1.26 | 0.615 | 1.25 | 0.210 | 0.734 |
| Trimethylamine | 0.89 | 0.67 | 0.505 | 0.623 | 0.83 | 0.866 | 0.91 | 0.500 | 0.704 |
| Cadaverine | 1.36 | 0.27 | 0.788 | 0.814 | 1.55 | 0.628 | 1.22 | 0.943 | 0.661 |
| Tyramine | 0.41 | -0.33 | 0.740 | 0.791 | 0.35 | 0.563 | 0.74 | 0.910 | 0.579 |
| Putrescine | 0.20 | -2.14 | 0.035 | 0.103 | 0.14 | 0.013 | 0.27 | 0.567 | 0.084 |
| N-Acetylputrescine | 0.11 | -2.19 | 0.031 | 0.098 | 0.02 | 0.064 | 0.21 | 0.234 | 0.428 |
| Glucose | 0.38 | -3.91 | 0.000 | 0.003 | 0.36 | 0.008 | 0.38 | 0.009 | 0.446 |
| Galactose | 0.61 | -2.66 | 0.009 | 0.048 | 0.79 | 0.538 | 0.51 | 0.005 | 0.167 |
| Arabinose | 0.37 | -1.98 | 0.050 | 0.127 | 0.39 | 0.016 | 0.37 | 0.536 | 0.167 |
| Xylose | 0.38 | -3.31 | 0.001 | 0.012 | 0.59 | 0.124 | 0.32 | 0.004 | 0.348 |
| Ribose | 0.67 | -2.39 | 0.019 | 0.071 | 0.74 | 0.260 | 0.62 | 0.034 | 0.748 |
| Fucose | 0.54 | -0.61 | 0.543 | 0.623 | 0.56 | 0.626 | 0.52 | 0.711 | 0.882 |
| hexose-phosphate | 2.02 | 3.37 | 0.001 | 0.012 | 4.40 | 0.001 | 1.38 | 0.139 | 0.101 |
| Myo-Inositol | 0.48 | -1.96 | 0.053 | 0.127 | 0.03 | 0.090 | 0.62 | 0.069 | 0.936 |
| Deoxycholate | 0.44 | -3.28 | 0.001 | 0.012 | 0.55 | 0.229 | 0.36 | 0.002 | 0.309 |
| Lithodeoxycholate | 0.01 | -4.02 | 0.000 | 0.003 | 0.01 | 0.018 | 0.01 | 0.004 | 0.994 |
| Cholate | 0.13 | -5.06 | 0.000 | 0.000 | 0.13 | 0.036 | 0.14 | 0.000 | 0.358 |
| Alanine | 0.70 | -1.85 | 0.068 | 0.145 | 0.43 | 0.012 | 0.90 | 0.767 | 0.067 |
| Asparagine | 0.64 | -1.29 | 0.199 | 0.306 | 0.35 | 0.197 | 0.82 | 0.533 | 0.348 |
| Aspartate | 0.83 | -1.88 | 0.063 | 0.145 | 0.50 | 0.008 | 0.98 | 0.560 | 0.152 |
| Citrulline | 0.90 | -0.60 | 0.550 | 0.623 | 0.87 | 0.765 | 0.91 | 0.562 | 0.952 |
| Glutamate | 0.84 | -1.34 | 0.184 | 0.299 | 0.94 | 0.975 | 0.77 | 0.059 | 0.255 |
| Glutamine | 0.67 | -4.24 | 0.000 | 0.002 | 0.60 | 0.003 | 0.72 | 0.005 | 0.579 |
| Glycine | 0.67 | -2.32 | 0.022 | 0.074 | 0.57 | 0.022 | 0.73 | 0.247 | 0.353 |
| Histidine | 0.78 | -2.45 | 0.016 | 0.064 | 0.72 | 0.111 | 0.82 | 0.079 | 0.876 |
| Urocanate | 0.72 | -1.28 | 0.204 | 0.306 | 0.62 | 0.843 | 0.79 | 0.123 | 0.441 |
| Isoleucine | 0.70 | -3.01 | 0.003 | 0.021 | 0.68 | 0.067 | 0.71 | 0.025 | 0.853 |
| Leucine | 0.82 | -1.86 | 0.066 | 0.145 | 0.85 | 0.373 | 0.80 | 0.095 | 0.813 |
| Lysine | 0.81 | -1.81 | 0.073 | 0.152 | 0.84 | 0.243 | 0.79 | 0.183 | 0.859 |
| Glutarate | 1.05 | 0.62 | 0.538 | 0.623 | 1.40 | 0.389 | 0.90 | 0.990 | 0.473 |
| 5-Aminovalerate | 0.59 | -1.28 | 0.205 | 0.306 | 0.54 | 0.502 | 0.64 | 0.284 | 0.807 |
| 2-Piperidinone | 0.22 | -1.15 | 0.253 | 0.366 | NA | 0.083 | 0.67 | 0.937 | 0.206 |
| Methionine | 0.79 | -1.88 | 0.063 | 0.145 | 0.79 | 0.239 | 0.79 | 0.161 | 0.929 |
| Ornithine | 0.59 | -3.33 | 0.001 | 0.012 | 0.53 | 0.006 | 0.65 | 0.059 | 0.328 |
| Phenylalanine | 0.81 | -1.97 | 0.051 | 0.127 | 0.80 | 0.302 | 0.82 | 0.096 | 0.883 |
| Proline | 0.85 | -1.13 | 0.262 | 0.373 | 0.94 | 0.808 | 0.76 | 0.206 | 0.511 |
| Serine | 0.72 | -2.52 | 0.014 | 0.061 | 0.60 | 0.030 | 0.81 | 0.160 | 0.424 |
| Taurine | 0.39 | -4.59 | 0.000 | 0.001 | 0.43 | 0.027 | 0.37 | 0.000 | 0.497 |
| Threonine | 0.80 | -2.04 | 0.044 | 0.119 | 0.78 | 0.296 | 0.81 | 0.060 | 0.806 |
| Tryptophan | 0.76 | -2.46 | 0.016 | 0.064 | 0.74 | 0.095 | 0.77 | 0.088 | 0.739 |
| Tyrosine | 0.76 | -2.34 | 0.021 | 0.074 | 0.75 | 0.191 | 0.77 | 0.059 | 0.936 |
| Valine | 0.82 | -1.66 | 0.100 | 0.195 | 0.88 | 0.467 | 0.79 | 0.131 | 0.701 |
| Methylsuccinate | 1.21 | 0.46 | 0.645 | 0.705 | 1.48 | 0.980 | 1.12 | 0.527 | 0.684 |
| 3-Methyl-2-oxovalerate | 0.79 | -1.42 | 0.160 | 0.274 | 0.93 | 0.864 | 0.63 | 0.080 | 0.357 |
| N6-Acetyllysine | 0.48 | -1.71 | 0.091 | 0.182 | 0.52 | 0.094 | 0.43 | 0.452 | 0.372 |
| methylamino acid | 0.71 | -2.35 | 0.021 | 0.074 | 0.81 | 0.232 | 0.68 | 0.011 | 0.496 |
| Succinate | 1.34 | -1.38 | 0.170 | 0.280 | 0.16 | 0.043 | 1.92 | 0.915 | 0.128 |
| Pyruvate | 0.83 | -1.44 | 0.153 | 0.272 | 0.73 | 0.180 | 0.90 | 0.491 | 0.443 |
| Fumarate | 0.98 | -0.80 | 0.426 | 0.563 | 0.70 | 0.185 | 1.17 | 0.928 | 0.258 |
| Malate | 0.60 | -1.40 | 0.165 | 0.278 | NA | 0.330 | 0.61 | 0.098 | 0.300 |
| 1,3-Dihydroxyacetone | 0.88 | -0.01 | 0.993 | 0.996 | 0.58 | 0.433 | 1.10 | 0.412 | 0.243 |
| Malonate | 0.81 | -1.65 | 0.102 | 0.196 | 0.44 | 0.070 | 1.13 | 0.464 | 0.458 |
| 3-Hydroxybutyrate | 1.88 | 0.60 | 0.548 | 0.623 | 5.78 | 0.411 | 0.67 | 0.982 | 0.452 |
| Acetone | 1.63 | 0.70 | 0.488 | 0.611 | 0.41 | 0.533 | NA | 0.023 | 0.082 |
| Glycerol | 0.68 | -3.20 | 0.002 | 0.015 | 1.11 | 0.783 | 0.47 | 0.000 | 0.003 |
| Isopropanol | 1.07 | 2.00 | 0.048 | 0.126 | 0.73 | 0.885 | 1.51 | 0.010 | 0.152 |
| Propylene glycol | 1.21 | 1.08 | 0.281 | 0.395 | 1.75 | 0.552 | 0.86 | 0.049 | 0.087 |
| Choline | 0.67 | -1.47 | 0.145 | 0.266 | 0.65 | 0.674 | 0.72 | 0.080 | 0.537 |
| Dimethylglycine | 0.61 | -0.59 | 0.557 | 0.623 | 0.55 | 0.425 | 0.64 | 0.953 | 0.519 |
| Hypoxanthine | 0.83 | -1.00 | 0.322 | 0.438 | 0.93 | 0.909 | 0.77 | 0.118 | 0.324 |
| Uracil | 0.79 | -1.43 | 0.156 | 0.272 | 0.85 | 0.623 | 0.75 | 0.151 | 0.629 |
| β-Alanine | 0.35 | -4.13 | 0.000 | 0.002 | 0.30 | 0.009 | 0.46 | 0.001 | 0.597 |
| Uridine | 0.50 | 0.03 | 0.973 | 0.994 | 0.24 | 0.346 | 0.66 | 0.410 | 0.209 |
| Xanthine | 0.81 | -1.30 | 0.198 | 0.306 | 0.83 | 0.382 | 0.80 | 0.356 | 0.919 |
| Cytidine | 0.44 | -1.28 | 0.204 | 0.306 | 0.51 | 0.470 | 0.37 | 0.299 | 0.999 |
| Guanosine | 0.21 | -3.16 | 0.002 | 0.018 | NA | 0.005 | 0.40 | 0.191 | 0.042 |
|  |  |  |  |  |  |  |  |  |  |
| Inosine | 0.80 | 0.70 | 0.486 | 0.611 | 0.88 | 0.190 | 0.74 | 0.867 | 0.255 |
| Niacinamide | 1.03 | 1.00 | 0.321 | 0.438 | 0.39 | 0.887 | NA | 0.085 | 0.296 |
| Nicotinate | 0.79 | -1.72 | 0.088 | 0.180 | 0.74 | 0.162 | 0.84 | 0.314 | 0.593 |

NA – Insufficient data to estimate ratio.
